# 2139. High Rates of Non-Susceptibility to Common Oral Antibiotics Among *Streptococcus pneumoniae* Clinical Isolates from the United States (2019-2021)

**DOI:** 10.1093/ofid/ofad500.1762

**Published:** 2023-11-27

**Authors:** Lalitagauri M Deshpande, Michael D Huband, Sarah Charbon, Mariana Castanheira, Rodrigo E Mendes

**Affiliations:** JMI Laboratories, North Liberty, Iowa; JMI Laboratories, North Liberty, Iowa; JMI Laboratories, North Liberty, Iowa; JMI Laboratories, North Liberty, Iowa; JMI Laboratories, North Liberty, Iowa

## Abstract

**Background:**

Major guidelines recommend β-lactams, macrolides, doxycycline (DOX) or respiratory quinolone monotherapy for outpatients with non-severe community-acquired bacterial pneumonia (CABP). Combination therapy is recommended for outpatients with comorbidities. Omadacycline (OMC) was equivalent to moxifloxacin as monotherapy for adults with CABP and was approved for CABP by the US FDA (2018). The activity of OMC and comparators was evaluated against *S. pneumoniae* (SPN) from the USA, as well as the resistance (R) mechanisms in tetracycline (TET) non-susceptible (NS) SPN.

**Figure:**
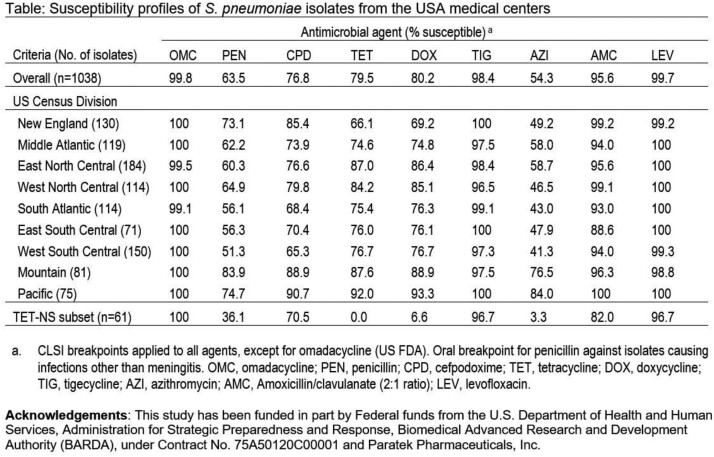

**Methods:**

1,038 SPN from 31 USA centers (2019-2021) were tested by CLSI broth microdilution. Isolates were from CABP (933), bloodstream infection (72), or nosocomial pneumonia (33). A selection of 119 SPN with DOX MIC of 0.25 - >8 mg/L were subjected to genome sequencing for screening of acquired TET-R genes and target site mutations, and serotyping (ST).

**Results:**

Most SPN isolates were susceptible (S) to OMC (99.8%; FDA), levofloxacin (LEV, 99.7%; CLSI) and tigecycline (TIG; 98.4%; FDA). Other agents had limited activity overall (penicillin 63.5%; or DOX 80.2%; or azithromycin 54.3%) with decreased S against isolates from certain US regions (Table). Only OMC (100% S), LEV (96.8% S) and TIG (96.7% S) were active against TET-NS (MIC, ≥2 mg/L) SPN. Cefpodoxime (70.5% S) and amoxicillin-clavulanate (82.0% S) had suboptimal activity against this R subset and other agent had S ≤36.1%. Isolates from the Pacific region tended to be more S than those from other regions. All TET-NS and 1 TET-S (MIC, 1 mg/L) isolates carried *tet*(M), and TET-NS had low S to DOX (6.6% S) and azithromycin (3.3% S). TET target site mutations were not observed. SPN belonged to 21 ST; 22F and 35B were the most common among TET-S SPN, whereas 15A, 19A, 09N, and 23A were common among TET-NS SPN.

**Conclusion:**

Options recommended for the empiric treatment of outpatients with CABP show low S against SPN from the US, except against isolates from the Pacific region. Tet(M) remains the dominant R mechanism, which did not affect OMC activity but significantly altered DOX activity. These data suggest that OMC represents a potential empiric option for treating pneumonia caused by SPN in the USA, including against isolates carrying *tet*(M).

**Disclosures:**

**Lalitagauri M. Deshpande, PhD**, Melinta: Grant/Research Support|Paratek: Grant/Research Support **Michael D. Huband, BS**, BARDA: This study has been funded in part by BARDA under Contract No. 75A50120C00001.|Entasis: Grant/Research Support|Paratek: Grant/Research Support|Pfizer: Grant/Research Support **Sarah Charbon, MHS, MLS(ASCP)CM**, Paratek: Grant/Research Support **Mariana Castanheira, PhD**, AbbVie: Grant/Research Support|Basilea: Grant/Research Support|bioMerieux: Grant/Research Support|Cipla: Grant/Research Support|CorMedix: Grant/Research Support|Entasis: Grant/Research Support|Melinta: Grant/Research Support|Paratek: Grant/Research Support|Pfizer: Grant/Research Support|Shionogi: Grant/Research Support **Rodrigo E. Mendes, PhD**, AbbVie: Grant/Research Support|Basilea: Grant/Research Support|Cipla: Grant/Research Support|Entasis: Grant/Research Support|GSK: Grant/Research Support|Paratek: Grant/Research Support|Pfizer: Grant/Research Support|Shionogi: Grant/Research Support

